# Comparative Analysis of Guatemalan and Qing Dynasty Jadeite Elemental Signs

**DOI:** 10.3390/molecules28073119

**Published:** 2023-03-31

**Authors:** Xinxin Liu, Qishen Zhou, Yanlin Wang, Jun Shu, Shaokui Pan, Fangmin Zhan

**Affiliations:** 1 School of gemology, China University of Geosciences (Wuhan), Wuhan 430074, China; 1202121285@cug.edu.cn (X.L.); zqs@cug.edu.cn (Q.Z.); wyl1202111015@cug.edu.cn (Y.W.); skpan@cug.edu.cn (S.P.); zfmwcy@yeah.net (F.Z.); 2 Hubei Key Research Base of Humanities and Social Sciences—Center for Jewelry Heritage and Innovation, Wuhan 430301, China

**Keywords:** Guatemalan jadeite, Qing dynasty jadeite, La-ICP-MS, element

## Abstract

Different jadeites have different characteristics. In this paper, the La-ICP-MS test is used to compare and analyze the elemental characteristics of jadeite in Guatemala and the Qing dynasty. The test results show that the highest value of Guatemalan jadeite Ca can reach 2.5 apfu, while the highest value of Qing dynasty jadeite is 0.73 apfu. The highest value of Na is the same for both. The concentration distribution range and highest value of Guatemalan jadeite and Qing dynasty jadeite Mg/(Mg + Fe) are the same. Guatemalan jadeite and Qing dynasty jadeite have a very wide content of trace elements. Qing dynasty Ca/(Mg + Fe) distribution is wider. Concentrations of Guatemalan and Qing dynasty jadeite Sr/Ba, which is a marine sediment, are greater than 1. The Ba in the Qing dynasty jadeite sediments contains a large amount of clay, resulting in higher levels than the average amount in Guatemalan jadeite Ba. The standard distribution map is similar, showing a “horn” shape. The Sr distribution is uneven. Guatemalan jadeite is heavily enriched in rare earths. Eu shows positive and negative abnormalities. The total rare earth value is 8.15 ppm. Qing Dynasty jadeite shows light rare earth enrichment, and Eu is a positive anomaly. The total rare earth value is 7.07 ppm. The characteristics of the two elements are somewhat similar, but different, which does not rule out the possibility that Qing dynasty jadeite came from Guatemala.

## 1. Introduction

Because jadeite grows in a special environment with high pressure and low temperature, it is only produced in a few countries around the world [1,2,3,4]. At present, there is no precise location from which Qing dynasty jadeite was sourced. Guatemala is a commercial producer of jadeite. Guatemala’s jadeite production in the market is large. Guatemalan jadeite is the closest in appearance and color to Qing dynasty jadeite, with the difference not being distinguishable using conventional gemological instruments. There are some differences between European and Qing dynasty jadeite; with jade deposits similar to Qing dynasty jadeite having been found only in the United States in 1939 and in Russia in 1959. The quality of Guatemalan and Qing dynasty jadeite is similar, with a large number of flat jadeite objects found in artefacts excavated from the Yama culture, and jadeite deposits were strictly guarded after Guatemala became a colony [1], whereas there is no clear evidence that it was not possible to mine jadeite in Guatemala during the Qing dynasty. Therefore, it is possible that Qing dynasty jadeite came from Guatemala [5]. Therefore, distinguishing the characteristics of Qing jadeite from those of Guatemalan jadeite is an important topic.

Chemical composition analysis can be used to classify jadeite mineral types and explore the distribution of different sources [6,7,8,9]. Yu Zhang believes that the Ca, Mg, and Fe levels are higher in Guatemalan jadeite than in Burmese jadeite [10]. According to the research of trace elements and rare earth elements of jadeite ore, the similarity, ore type, color mechanism, and other phenomena of jadeite can be understood, and it is also possible to explore its mineral-forming source and mineralization environment, meaning that jadeite prospecting will play an extremely important role [11]. Because jadeite is always considered to be P-type or R-type, the trend of trace elements is the best way to study jadeite rock [12,13,14]. The enrichment of trace elements varies from place to place. The degree of enrichment of Zr and Hf at different points of origin is different [15,16,17]. In Guatemala, jadeite rocks have been found to exhibit Ba enrichment and Sr distribution that can be classified as uneven partial enrichment and partial loss, and these features can also be seen in eclogite [14]. The REE distribution pattern of jadeite rock is an inverted U shape, and the degree of depression is different for different points of origin [18,19,20,21]. As an increasing amount of jadeite from Guatemala has appeared on the market, trace elements and rare earth elements have been analyzed in Guatemala. However, no research has been reported on Qing dynasty jadeite. In this paper, the ICP test is used to analyze the chemical composition, trace elements, and rare earth elements of Guatemalan and Qing jadeite in order to study the differences between the two.

## 2. Results

### 2.1. Main Chemical Components

Jadeite is the main mineral component of jadeite, while the main chemical components are SiO_2_, Al_2_O_3_, and Na_2_O. The theoretical values of SiO_2_, Al_2_O_3_, and Na_2_O content in pure jadeite are 59.4 wt%, 25.2 wt%, and 15.4 wt%, respectively [22]. The chemical composition of Guatemalan jadeite consists primarily of SiO_2_, Al_2_O_3_, and Na_2_O. SiO_2_ is present in the highest content among them. The content of SiO_2_ is 53.7–66.3 wt%, with an average value of 57.3 wt%; the content of Al_2_O_3_ is 11.1–28.1 wt%, with an average value of 20.6 wt%; and the content of Na_2_O is 7.34–16.0 wt%, with an average value of 13.6 wt%, which is close to the theoretical value. Fifteen points are determined to be omphacite, while the remaining points are determined to be jadeite (Table 1). This suggests that the primary compositions of the different Guatemalan samples differ from one another. The FeO content in W13-3 reaches up to 9.22 wt%, while the CaO/MgO ratio reaches 6.50, and the content of omphacite here is high. Otherwise, the CaO/MgO ratio for the other samples relatively stable, basically in the range 1.22–1.92.

The chemical composition of Qing dynasty jadeite mainly consists of SiO_2_, Al_2_O_3_, and Na_2_O. SiO_2_ is present in the highest content among them. The SiO_2_ content is 56.7–63.3 wt%, with an average value of 59.4 wt%; the content of Al_2_O_3_ is 6.8–24.7 wt%, with an average value of 22.3 wt%; and the content of Na_2_O is 9.8–15.4 wt%, with an average value of 13.6 wt%, which is close to the theoretical value. One point is determined to be omphacite, while the other points are determined to be jadeite (Table 2), indicating that the main component of Qing jadeite is jadeite, and there is a small amount of omphacite. The CaO/MgO ratio in Qing dynasty jadeite is generally lower than in Guatemalan jadeite, where the lowest ratio is 0.22, and the highest ratio is 1.59.

**Figure 1:**
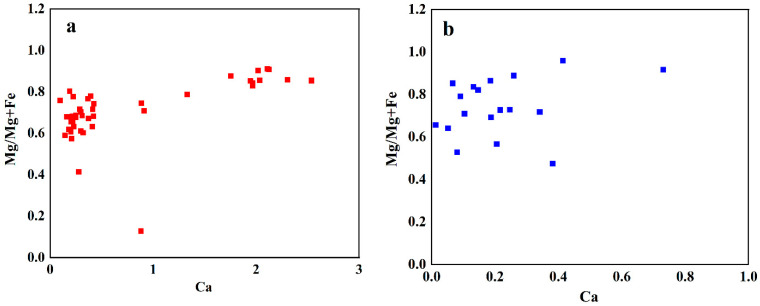
Discriminant diagram of Ca-Mg/(Mg + Fe) in Guatemalan and Qing dynasty jadeite. (**a**) Discriminant diagram of Guatemalan jadeite; (**b**) discriminant diagram of Qing dynasty jadeite. Each element is represented by an apfu value. apfu: Atoms per formula unit.

The discriminant chart for Ca-Mg/(Mg + Fe) of Guatemalan and Qing dynasty jadeite is presented in Figure 1. The highest value of Ca in Guatemalan jadeite reaches 2.5 apfu, while the lowest reaches 0.09 apfu. For the Ca of Qing dynasty jadeite, the highest value obtained is 0.73 apfu, while the lowest is 0.01 apfu. Pyroxene content increases with increasing Ca in jadeite [23]; therefore, it can be inferred that the Ca content in Guatemalan jadeite is greater than that of Qing dynasty jadeite. The highest value obtained for Mg/(Mg + Fe) is 0.91 apfu in Guatemalan jadeite, while the lowest is 0.91 apfu. The value of Mg/(Mg + Fe) is concentrated in the range 0.6–0.9 apfu. The highest value of Ca in Qing dynasty jadeite reaches 0.73 apfu, while the lowest reaches 0.52 apfu. The value of Mg/(Mg + Fe) is concentrated in the range0.6–0.9 apfu. Therefore, the concentrated distribution range and the highest value of Mg/(Mg + Fe) are the same in Guatemalan jadeite and Qing dynasty jadeite.

**Figure 2:**
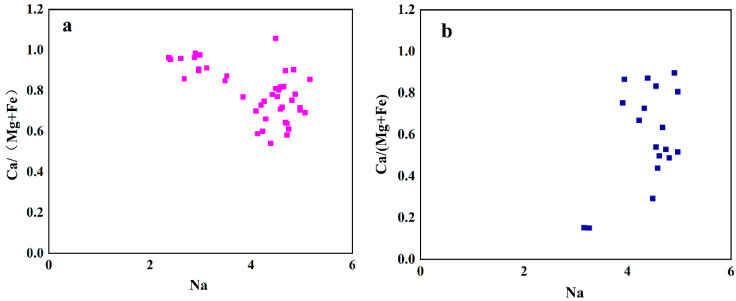
Discriminant diagram of Na-Ca/(Mg + Fe) in Guatemalan and Qing dynasty jadeite. (**a**) Discriminant diagram of Guatemalan jadeit; (**b**) discriminant diagram of Qing dynasty jadeite. Each element is represented by an apfu value.

The discriminant diagram for Na-Ca/(Mg + Fe) of Guatemalan and Qing dynasty jadeite is presented in Figure 2. The highest value of Na in Guatemalan jadeite reaches 5.1 apfu, while the lowest reaches 0.09 apfu, with the value of Na being concentrated in the range 4–5 apfu. The highest value of Na in Qing dynasty jadeite reaches 5.1 apfu, while the lowest reaches 3.1 apfu, with the value of Na being concentrated in the range 4–5 apfu. The highest values of Na are the same in Guatemalan jadeite and Qing dynasty jadeite at 5.1 apfu, and are concentrated in the range 4–5 apfu. The highest value of Ca/(Mg + Fe) in Guatemalan jadeite reaches 0.97 apfu, while the lowest value is 0.54 apfu, with the values being concentrated in the range 0.5–1.0 apfu. The highest value of Ca/(Mg + Fe) in Qing dynasty jadeite reaches a value of 0.90, while the lowest value reaches 0.15, with those values being concentrated in the range 0.5–0.9 apfu in Qing jadeite. The distribution range of Ca/(Mg + Fe) in the Qing dynasty jadeite is wider.

### 2.2. Trace Elements

**Figure 3:**
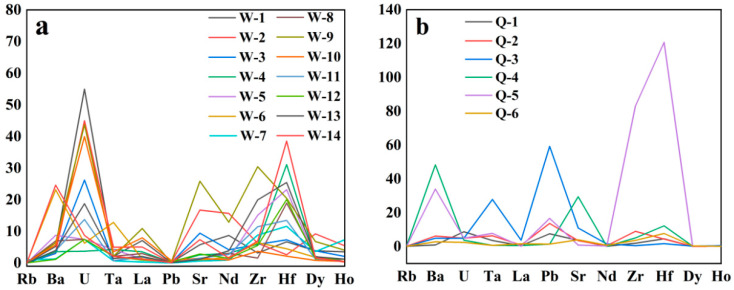
Map of standard levels of trace elements in Guatemalan and Qing dynasty jadeite. (**a**) is Guatemalan jadeite and (**b**) is Qing dynasty jadeite.

The contents of trace elements in Guatemalan and Qing dynasty jadeite are shown in Table 3 and Table 4. The content ratio of Sr/Ba, which is a marine sediment, is greater than 1 in both Guatemalan and Qing dynasty jadeite. The average content of Ba in Guatemalan jadeite is 17.5 ppm. The average content of Ba in Qing Dynasty jadeite is 39.10 ppm. The Ba in the Qing dynasty jadeite sediment contains a large amount of clay, resulting in higher content [24]. The average value of Zr/Hf in Guatemalan jadeite is 31.39 ppm, which is close to the Zr/Hf ratio of chondrites. The average value of Zr/Hf in Qing dynasty jadeite is 25.09 ppm, which is less than the Zr/Hf ratio of chondrites [25]. Using the data proposed by Sun and W.F. McDonough to standardize the trace element map (Figure 3) [26], the trace element content of Guatemalan jadeite and Qing dynasty jadeite can be observed to have a very wide range, while the distribution map is similar, showing a “horn” shape. The Sr distribution is uneven. Only one sample of Guatemalan jadeite was enriched with Zr, while Guatemalan jadeite had the largest number of samples enriched with Hf. Qing dynasty jadeite, apart from sample 6, was enriched with Ba, while one sample was enriched with Zr, and five samples were enriched with Hf.

### 2.3. Rare Earth Elements

**Figure 4:**
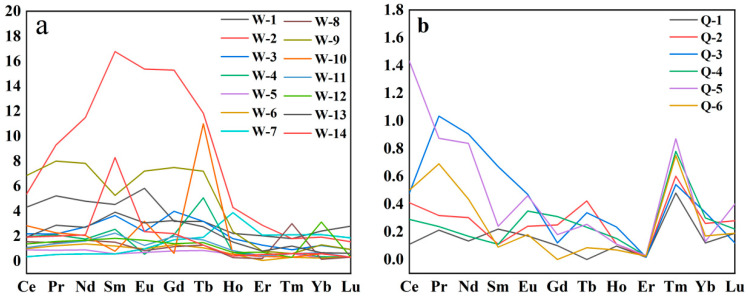
Standard content map of rare earth elements in Guatemalan and Qing dynasty jadeite. (**a**) is Guatemalan jadeite and (**b**) is Qing dynasty jadeite.

Rare earth element contents are provided in Table 5 and Table 6, standardized using the data obtained by Masuda [27]. The distribution of rare earth elements in Guatemalan jadeite has the following characteristics: the (La/Yb)_N_ content ratios of samples 7 and 12 are smaller than 1 and belong to the loss type. The (La/Yb)_N_ ratios of the other samples are greater than 1 and belong to the enrichment type. The average total rare earth value is 8.15 ppm. The average mean of LREE/HREE is 2.72, showing that the samples are heavily enriched with rare earth; samples 1, 5, 9, 10, and 12 possess an Eu/Eu* ratio greater than 1, thus representing positive europium anomalies, while the other samples are negative abnormalities. According to the distribution pattern diagram of Guatemalan jadeite: the distribution mode of samples 4–7 and 11–12 remains relatively stable, while sample 9 is concave in a clear “U” shape and tilted to the right (Figure 4a).

The distribution characteristics of rare earth elements in Qing dynasty jadeite have the following characteristics: values of (La/Yb)_N_ greater than 1 indicate samples belonging to the enrichment type. The average total amount of rare earths is 7.07 ppm. The average LREE/HREE is 7.55, indicating light rare earth enrichment. Due to the low content of rare earth elements, the content of Gd elements was not measured for sample 6; therefore, it is replaced with Yb for calculation purposes. Eu/Eu* values greater than 1 indicate positive europium anomalies, implying a relationship with feldspar. According to the rare earth element distribution map of the Qing dynasty jadeite, the distribution pattern of Qing dynasty jadeite shows a slight “U”-shaped downward concavity, and the curve slopes to the right (Figure 4b).

## 3. Materials and Methods

**Figure 5:**
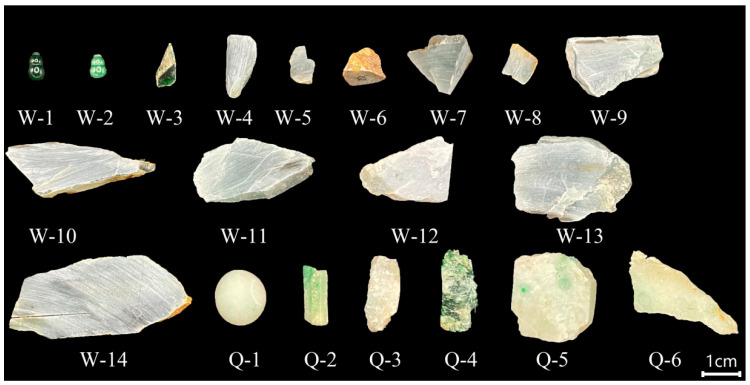
Guatemalan and Qing dynasty jadeite samples. (W-1 and W-2: cut thinly, use water transfer process, and show obvious green with tin foil back cover; W-3: white leather corners (jade peel visible), the color is darker and has a large number of black inclusions; W-4: ice jadeite interface material. The structure is dense, the transparency is good, and there is white cotton; W-5: the skin is thin and evenly distributed, and the inner jade flesh is delicate in texture and relatively high in transparency; W-6: the surface is infected with yellow and the structure is loose; W-7: the water is good, there are many cracks, and the skin shell is an incomplete thin layer of white; W-8: porcelain land, the germ quality is poor, but the jade is still relatively delicate to the naked eye; W-9: contains veined green veins, with shear fissures and goose-like fissures; W-10: the cortex is thin, and there are fine veins of red limonite on the surface of the rough, which are flush to the rough along the cracks; W-11: contains fine vein-like green veins, and the water is excellent; W-12: there are red limonite fine vein fractures on the surface, no skin shell, and the surface is evacuated; W-13: rough or standing to the touch, the fog under the skin shell (semi-weathered jadeite layer) is thin, white mist, dense in texture, green pine flowers on the epidermis, and finely veined green inside; W-14: there is a black–green–yellow–gray–blue color transition, and white cotton is visible in the gray–blue part. Q-1: contains small fine veins of green, with presence of a fly wing phenomenon; Q-2: the whole is green, the fly wing phenomenon is present, and there are cracks on the surface; Q-3: There is a small green vein on the surface and cracks on the surface; Q-4: the color is darker, there are cracks on the surface, and there are black inclusions; Q-5: contains green veins, there are many cracks on the surface, the fly wing phenomenon is present; Q-6: the surface has green veins, brown substance, and the fly wing phenomenon is obvious.)

In this experiment, 20 samples were selected, of which W-1–W-14 were Guatemalan jadeite. They were purchased from Sihui Guatemalan rough jadeite suppliers. The colors were gray-blue, green, and yellow-brown. Q-1–Q-6 were samples of Qing dynasty jadeite purchased from Tengchong, Yunnan. The color were colorless, green, and gray-green. The samples are shown in Figure 5.

The sample sizes were suitable for being placed in the sample chamber, and the sample surface was wiped clean with alcohol before the experiment. The element content tests of the Guatemalan and Qing dynasty jadeite were carried out using LA-ICP-MS (this comprises two sets of equipment used together, where LA refers to laser equipment, and ICP-MS refers to compositional molecular instruments) in Agilent 7900. As trace element calibration standard samples, NIST 610, BHVO-2G, BIR-1G, and BCR-2G were used. For all of the above samples, the GeoLas HD laser ablation system was used for substance analysis, and plasma mass spectrometry was performed using an Agilent 7900, where a laser energy of 80 mJ, a frequency of 5 Hz, and a laser beam spot diameter of 44 μm were used.

## 4. Conclusions

The Guatemalan and Qing dynasty jadeites primarily exhibit chemical compositions that are similar. The compositions of different samples of Guatemalan jadeite differ from one another. However, the main component of Qing dynasty jadeite is jadeite, and there is a small amount of omphacite. The CaO/MgO value of Qing dynasty jadeite is generally lower than that of Guatemalan jadeite. The scattered distribution has the following characteristics: the content of jadeite pyroxene increases with increasing Ca; therefore, the content of Ca in Guatemalan jadeite can be concluded to be greater than that in Qing dynasty jadeite. The highest value of Na is the same for both. The concentrated distribution range and highest value of Mg/(Mg + Fe) are the same for Guatemalan jadeite and Qing dynasty jadeite, while the distribution of Guatemalan jadeite is more concentrated. The distribution of Ca/(Mg + Fe) values in Qing dynasty jadeite is wider.

The values of Sr/Ba, which is a marine sediment, are greater than 1 in Guatemalan and Ming and Qing dynasty jadeite. The Ba in Qing Dynasty jadeite sediments contained a large amount of clay, resulting in a higher content. The Zr/Hf ratio in Guatemalan jadeite is close to that of chondrite meteorite. However, the ratio of Zr/Hf in Qing dynasty jadeite is lower than that of chondrite Zr/Hf. Guatemalan jadeite and Qing dynasty jadeite have a very wide range of trace element content, with a similar standard distribution map, which shows a “horn” shape. Guatemalan jadeite is heavily enriched with rare earths, and Eu shows positive and negative anomalies. The total content of rare earth is 8.15 ppm. Qing dynasty jadeite exhibits light rare earth enrichment, while Eu represents a positive anomaly. The total content of rare earth is 7.07 ppm. The characteristics of the two elements are somewhat similar, but different, which does not rule out the possibility that Qing dynasty jadeite originally came from Guatemalan. Next, we will also study the differences between Qing dynasty jadeite and jadeite from other sources.

## Figures and Tables

**Table 1:** Main chemical composition of Guatemalan jadeite (wt%).

	SiO_2_	Al_2_O_3_	Na_2_O	FeO	CaO	MgO	CaO/MgO	Na/Na + Ca	
W-1-1	54.90	11.10	7.34	2.74	14.2	9.08	1.56	0.34	omphacite
W-1-2	55.00	12.70	8.08	2.45	12.9	8.31	1.55	0.39	omphacite
W-1-3	54.60	11.10	7.47	2.80	14.2	9.13	1.56	0.35	omphacite
W-2-1	55.30	14.70	9.25	1.46	11.3	7.52	1.50	0.50	omphacite
W-2-2	55.00	14.20	8.93	1.46	11.9	8.07	1.47	0.43	omphacite
W-2-3	55.40	14.10	8.99	1.38	11.8	7.86	1.50	0.43	omphacite
W-3-1	55.70	14.10	9.19	2.28	10.9	7.45	1.46	0.46	omphacite
W-3-2	55.50	13.60	9.19	2.47	11.0	7.36	1.49	0.45	omphacite
W-3-3	56.30	15.30	9.67	1.72	9.83	6.79	1.45	0.50	omphacite
W-4-1	57.40	23.50	14.50	1.30	1.67	1.14	1.46	0.90	Jadeite
W-4-2	57.40	23.30	14.00	1.02	2.37	1.64	1.45	0.86	Jadeite
W-4-3	57.80	23.90	14.60	0.90	1.39	1.06	1.31	0.91	Jadeite
W-5-1	57.10	23.90	14.20	1.07	2.08	1.23	1.69	0.87	Jadeite
W-5-2	57.10	23.40	14.10	1.35	2.30	1.30	1.77	0.86	Jadeite
W-5-3	57.30	24.00	14.40	1.12	1.80	0.95	1.89	0.89	Jadeite
W-6-1	57.60	24.20	14.70	0.89	1.23	0.95	1.29	0.92	Jadeite
W-6-2	57.60	24.50	14.60	0.97	1.12	0.84	1.33	0.93	Jadeite
W-6-3	57.50	24.00	14.20	0.99	1.74	1.21	1.44	0.89	Jadeite
W-7-1	66.40	19.30	10.80	0.69	1.60	0.97	1.65	0.87	Jadeite
W-7-2	64.30	21.10	11.90	0.65	1.02	0.59	1.73	0.92	Jadeite
W-7-3	63.10	22.10	12.70	0.61	0.81	0.49	1.65	0.94	Jadeite
W-8-1	61.90	21.80	12.80	1.09	1.17	0.82	1.43	0.92	Jadeite
W-8-2	60.90	22.80	13.30	0.77	1.15	0.82	1.40	0.92	Jadeite
W-8-3	59.40	22.00	13.60	2.17	1.56	0.86	1.81	0.90	Jadeite
W-9-1	57.10	11.80	8.30	2.80	11.0	7.65	1.44	0.43	omphacite
W-9-2	56.60	16.90	10.90	2.34	7.45	4.83	1.54	0.59	omphacite
W-9-3	57.00	24.90	15.00	0.62	1.39	0.76	1.83	0.91	Jadeite
W-10-1	53.80	28.10	15.10	0.46	1.26	0.90	1.40	0.92	Jadeite
W-10-2	55.70	26.30	15.40	0.38	1.06	0.87	1.22	0.94	Jadeite
W-10-3	55.20	27.60	16.00	0.20	0.55	0.35	1.57	0.97	Jadeite
W-11-1	54.30	20.70	13.70	2.44	5.10	3.33	1.53	0.73	omphacite
W-11-2	53.90	21.10	13.00	2.22	4.96	3.65	1.36	0.72	omphacite
W-11-3	55.30	25.00	15.70	0.92	1.67	1.22	1.37	0.90	Jadeite
W-12-1	59.00	24.50	14.30	0.52	0.91	0.62	1.47	0.94	Jadeite
W-12-2	58.30	23.10	13.90	0.77	2.20	1.52	1.45	0.86	Jadeite
W-12-3	57.90	25.00	14.50	0.55	1.21	0.66	1.83	0.92	Jadeite
W-13-1	56.60	24.80	15.40	0.85	1.29	0.82	1.57	0.92	Jadeite
W-13-2	56.30	23.30	14.90	1.28	2.36	1.54	1.53	0.86	Jadeite
W-13-3	53.70	17.40	13.10	9.22	4.94	0.76	6.50	0.73	omphacite
W-14-1	59.40	11.50	7.43	2.22	11.4	7.36	1.55	0.39	omphacite
W-14-2	58.90	22.40	13.20	1.13	2.31	1.59	1.45	0.85	Jadeite
W-14-3	58.10	24.00	13.90	0.58	2.05	1.07	1.92	0.85	Jadeite

**Table 2:** Main chemical composition of Qing dynasty jadeite (wt%).

	SiO_2_	Al_2_O_3_	Na_2_O	FeO	CaO	MgO	CaO/MgO	Na/Na + Ca	
Q-1-1	63.10	23.10	13.10	0.20	0.29	0.20	1.45	0.98	Jadeite
Q-1-2	60.70	23.50	13.40	0.57	1.05	0.72	1.46	0.93	Jadeite
Q-1-3	59.40	23.80	14.10	0.51	1.21	0.76	1.59	0.92	Jadeite
Q-2-1	58.00	23.80	14.50	0.76	1.38	1.14	1.21	0.91	Jadeite
Q-2-2	57.60	26.10	14.70	0.41	0.58	0.56	1.04	0.96	Jadeite
Q-2-3	57.70	26.70	15.40	0.06	0.07	0.06	1.17	0.99	Jadeite
Q-3-1	57.80	25.30	15.20	0.21	0.82	0.54	1.52	0.95	Jadeite
Q-3-2	57.70	23.70	14.90	1.31	1.15	0.96	1.20	0.93	Jadeite
Q-3-3	57.70	25.80	15.40	0.17	0.51	0.36	1.42	0.97	Jadeite
Q-4-1	57.00	20.10	13.90	2.37	1.91	3.37	0.57	0.88	Jadeite
Q-4-2	56.90	22.00	14.30	2.90	2.14	1.47	1.46	0.87	Jadeite
Q-4-3	56.70	6.48	9.80	2.88	4.09	17.7	0.23	0.71	omphacite
Q-5-1	63.30	22.10	12.10	0.24	1.04	0.86	1.21	0.92	Jadeite
Q-5-2	62.70	22.20	12.20	0.24	1.45	1.07	1.36	0.89	Jadeite
Q-5-3	62.20	12.90	10.10	0.82	2.32	10.6	0.22	0.81	Jadeite
Q-6-1	60.70	24.20	13.60	0.18	0.74	0.51	1.45	0.95	Jadeite
Q-6-2	60.40	24.50	14.10	0.13	0.37	0.42	0.88	0.97	Jadeite
Q-6-3	59.30	24.90	14.20	0.62	0.45	0.39	1.15	0.97	Jadeite

**Table 3:** Guatemalan jadeite trace elements (ppm).

	W-1	W-2	W-3	W-4	W-5	W-6	W-7	W-8	W-9	W-10	W-11	W-12	W-13	W-14
Rb	0.04	0.02	0.07	0.21	0.08	0.15	2.31	0.24	0.06	0.48	0.40	0.17	0.13	0.06
Ba	12.60	8.40	7.30	8.70	21.10	55.80	3.10	16.80	15.40	13.90	9.50	2.80	9.70	59.40
U	0.44	0.36	0.21	0.03	0.06	0.05	0.06	0.06	0.35	0.32	0.11	0.06	0.15	0.07
Ta	0.03	0.07	0.01	0.06	0.06	0.18	0.01	0.03	0.03	0.05	0.01	0.03	0.02	0.03
La	1.69	1.20	0.46	0.84	0.31	0.32	0.08	0.71	2.59	1.90	0.49	0.22	0.49	0.29
Pb	0.43	0.40	0.21	0.46	0.28	0.68	0.12	0.58	0.39	2.11	0.13	0.98	0.27	0.36
Sr	42.10	121.50	68.70	18.80	7.40	6.60	4.70	9.70	188.00	11.10	11.20	20.90	8.70	53.60
Nd	4.07	7.34	1.96	1.43	0.66	0.85	0.43	1.45	6.02	0.46	1.28	0.69	1.80	0.73
Zr	12.24	24.12	22.83	23.18	58.45	24.94	34.19	6.15	117.96	14.41	44.38	21.30	77.19	26.95
Hf	0.71	0.28	0.78	3.30	2.46	0.48	1.23	2.01	2.17	0.23	1.43	2.13	2.70	4.09
Dy	0.98	2.35	0.98	0.46	0.26	0.46	0.92	0.53	1.73	0.22	0.31	0.36	0.51	0.42

**Table 4:** Qing dynasty jadeite trace elements (ppm).

	Q-1	Q-2	Q-3	Q-4	Q-5	Q-6
Rb	0.41	0.43	0.27	1.27	0.45	0.39
Ba	2.40	15.02	11.86	116.5	81.86	6.93
U	0.07	0.04	0.04	0.03	0.04	0.02
Ta	0.05	0.09	0.39	0.01	0.11	0.01
La	0.12	0.21	0.89	0.15	0.05	0.40
Pb	3.87	3.65	3.62	4.15	2.50	7.13
Sr	27.05	25.34	79.84	214.00	6.69	29.20
Nd	0.08	0.27	0.74	0.11	0.17	0.37
Zr	7.86	34.99	2.00	19.83	321.46	14.12
Hf	0.49	0.47	0.18	1.30	19.50	0.82
Dy	0.03	0.03	0.07	0.07	0.07	0.09

**Table 5:** Guatemalan jadeite rare earth elements (ppm).

	W-1	W-2	W-3	W-4	W-5	W-6	W-7	W-8	W-9	W-10	W-11	W-12	W-13	W-14
Ce	0.60	1.07	0.25	0.24	0.10	0.14	0.06	0.17	0.92	0.26	0.16	0.18	1.56	1.55
Pr	2.86	6.86	1.63	1.05	0.53	0.82	0.34	0.99	4.67	1.22	0.96	0.99	0.33	0.23
Nd	0.87	3.22	0.7	0.49	0.11	0.23	0.11	0.29	1.01	0.15	0.43	0.35	1.61	1.25
Sm	0.42	1.11	0.17	0.04	0.05	0.07	0.07	0.06	0.52	0.23	0.09	0.12	0.75	1.59
Eu	0.82	3.96	1.03	0.55	0.21	0.34	0.44	0.30	1.94	0.16	0.51	0.36	0.22	0.17
Gd	0.15	0.56	0.15	0.24	0.04	0.05	0.09	0.06	0.34	0.52	0.08	0.07	0.84	0.57
Tb	0.75	2.49	0.81	0.38	0.22	0.38	0.61	0.37	1.35	0.17	0.74	0.25	0.13	0.06
Ho	0.43	0.61	0.27	0.08	0.07	0.01	0.44	0.04	0.17	0.08	0.09	0.15	0.11	0.03
Er	0.06	0.06	0.03	0.02	0.02	0.01	0.07	0.10	0.02	0.01	0.02	0.01	0.17	0.11
Tm	0.50	0.40	0.26	0.07	0.05	0.05	0.44	0.03	0.27	0.12	0.14	0.65	0.04	0.02
Yb	0.09	0.05	0.03	0.01	0.01	0.01	0.06	0.01	0.03	0.01	0.01	0.01	0.14	0.12
Lu	0.09	0.05	0.03	0.01	0.01	0.01	0.06	0.01	0.03	0.01	0.01	0.01	0.02	0.01
(La/Yb)_N_	1.58	1.88	1.75	5.66	5.02	3.04	0.14	10.57	5.04	7.15	1.70	0.42	3.16	3.58
Eu/Eu*	1.54	0.96	0.62	0.24	1.02	0.77	0.98	0.63	1.15	4.59	0.59	1.04	0.86	0.55

Eu/Eu* = Eu_N_/(Sm_N_ × Gd_N_)^1/2^.

**Table 6:** Qing dynasty jadeite rare earth elements (ppm).

	Q-1	Q-2	Q-3	Q-4	Q-5	Q-6
Ce	0.09	0.34	0.39	0.23	1.17	0.40
Pr	0.02	0.04	0.12	0.03	0.10	0.08
Nd	0.08	0.18	0.54	0.10	0.50	0.26
Sm	0.04	0.02	0.13	0.02	0.05	0.02
Eu	0.01	0.01	0.03	0.03	0.03	0.01
Gd	0.03	0.07	0.03	0.08	0.05	0.00
Tb	0.00	0.02	0.02	0.01	0.01	0.01
Dy	0.02	0.02	0.05	0.06	0.06	0.06
Ho	0.01	0.01	0.02	0.01	0.01	0.01
Er	0.02	0.02	0.02	0.03	0.03	0.03
Tm	0.01	0.00	0.01	0.01	0.02	0.00
Yb	0.02	0.06	0.07	0.06	0.03	0.04
Lu	0.01	0.01	0.01	0.01	0.01	0.01
(La/Yb)_N_	2.97	1.81	5.50	1.24	20.01	4.95
Eu/Eu*	2.25	2.79	3.28	3.73	4.26	1.68

Eu/Eu* = Eu_N_/(Sm_N_ × Gd_N_)^1/2^.

## Data Availability

The basic data is reflected in the paper, and some other data can be obtained by contacting us.
